# Prevalence of asthma in Saudi adults: findings from a national household survey, 2013

**DOI:** 10.1186/s12890-015-0080-5

**Published:** 2015-07-28

**Authors:** Maziar Moradi-Lakeh, Charbel El Bcheraoui, Farah Daoud, Marwa Tuffaha, Hannah Kravitz, Mohammad Al Saeedi, Mohammed Basulaiman, Ziad A. Memish, Mohammad A. AlMazroa, Abdullah A. Al Rabeeah, Ali H. Mokdad

**Affiliations:** Institute for Health Metrics and Evaluation, University of Washington, 2301 Fifth Ave., Suite 600, Seattle, WA 98121 USA; Ministry of Health of the Kingdom of Saudi Arabia, Assadah, Al Murabba, Riyadh, 12613 Saudi Arabia

**Keywords:** Asthma, Prevalence, Asthma attack, Asthma management, Saudi Arabia

## Abstract

**Background:**

There are not enough data on the epidemiology of asthma in the Kingdom of Saudi Arabia (KSA). We analyzed data from a national household survey conducted in KSA in 2013 to estimate prevalence, associated risk factors and control measurements of asthma.

**Methods:**

The Saudi Health Interview Survey was a cross-sectional national multistage survey of 10,735 individuals aged 15 years or older. The survey included a detailed household questionnaire and a physical exam. We used self-reported clinical diagnosis of asthma to assess prevalence of asthma.

**Results:**

The prevalence of asthma in KSA was 4.05 % (95 % confidence interval [CI]: 3.54–4.62 %). Asthma was less frequent in individuals with higher education but higher in former smokers and obese individuals. Around 76.7 % of asthma patients (95 % CI: 70.6–82.0 %) experienced an asthmatic attack, and 61.6 % (95 % CI: 54.4–68.4 %) visited a hospital/emergency room because of asthma during the past year. Asthma attack was less frequent in older patients (odds ratio [OR] = 0.78, 95 %CI: 0.59–0.96 for each decade of life). Current use of medication for asthma was highly associated with asthma attacks (OR = 9.14, 95 % CI: 3.29–25.38). Asthma attack was also more frequent in individuals who were exposed to secondhand smoking (OR = 2.17, 95 %CI: 1.05–4.45) and those who were obese (OR = 3.01, 95 %CI: 1.34–6.78).

**Conclusion:**

Saudi Arabia has a relatively low prevalence of diagnosed asthma; however, many of the patients with known asthma do not have it under good control. Our study calls for programs to inform patients about the importance and proper means of controlling their condition. Implementing and monitoring of clinical guidelines can also help to improve asthma control among patients as well as identify undiagnosed cases.

## Background

Asthma is a major health issue worldwide. It is among the top 30 highest-burden diseases based on the Global Burden of Disease 2010 study (GBD 2010) [1].

In the Kingdom of Saudi Arabia (KSA), asthma ranks 19th in terms of disability-adjusted life years (DALYs) and 26th in deaths [1, 2]. There are reports on its increasing pattern in KSA [3].

Few epidemiological studies exist on asthma in KSA. The Saudi initiative for asthma (2012) stated that the prevalence of asthma in Saudi adults is unknown, and the overall prevalence in Saudi children ranges from 8 to 25 % in different studies [4].

Al-Frayh reported an increase in the prevalence of asthma from 8 % in 1986 in Jeddah and Riyadh (costal humid and desert dry, respectively) to 23 % in 1995 in Jizan and Hail (costal humid and desert dry, respectively) among schoolchildren aged 6 to 18 years. He also reported in another study a prevalence of 33.7 % in Hofuf (inland eastern), 17.7 % in Riyadh (inland central), and 14.1 % in Jaddah (western coastal) in 2002 among schoolchildren [3, 5, 6]. Hijazi (1998) reported a prevalence of 12.1 % among children aged 12 years in Jaddeh [7].

Prevalence of bronchial asthma was reported to be 14 % in individuals aged 11 years or older in rural areas of the Asir region [8]. Al-Ghobian reported a prevalence of 19.6 % for physician-diagnosed asthma among students aged 16 to18 in Riyadh during the 2009–2010 academic year [9].

KSA provides free health care to all citizens. Therefore, it is crucial to have accurate and updated health information for planning. To estimate the prevalence, associated risk factors and control measurements of asthma in Saudi adults, we analyzed data from a national household survey conducted in 2013.

## Methods

The Saudi Health Interview Survey was a cross-sectional national multistage survey of adult Saudi citizens. Adulthood in KSA is defined as 15 years of age or older. KSA had a population of 26.9 million in 2013, with 5.6 million non-nationals. The sample size was designed to ensure a confidence level of 95 %, with a margin of error of 0.05 and a baseline level of indicators of 0.5. A previous national study conducted by MOH yielded a design effect of 1.5 and a response rate of 85 %. Adjusting for the design effect and response rate, a sample size of 11,806 was selected and rounded to 12,000. The whole country was divided into 13 regions, and each region was divided into subregions and blocks. All regions were included, and a probability proportional to size method was used to randomly select subregions and blocks. Households were randomly selected from each block. A roster of household members was conducted and an adult aged 15 or older was randomly selected from the household to be surveyed. If the randomly selected adult was not present, our surveyors made another appointment to return, up to three times, before considering the household as a nonresponse.

The Saudi Ministry of Health and its Institutional Review Board (IRB) approved the study protocol. The University of Washington IRB deemed the study as IRB-exempt, since the Institute for Health Metrics and Evaluation received de-identified data for this analysis. Only those individuals who agreed to participate and consented after receiving all required information about the survey were entered into the study.

The survey included questions for socio-demographic characteristics, self-report of clinician-diagnosed asthma and other chronic diseases, risk factors and functional status.

Individuals were classified into three groups—never smokers, former smokers and current smokers—through two questions: “Have you ever smoked any tobacco products, such as cigarettes, cigars or pipes or Shisha?” and “Do you currently smoke any tobacco products, such as cigarettes, cigars, pipes or Shisha?” To assess exposure to secondhand smoke, respondents were asked, “During the past 7 days, on how many days did someone in your home smoke when you were present?” and “During the past 7 days, on how many days did someone smoke in closed areas in your workplace or school (in the building, in a work area or a specific office) when you were present?” Those who reported such exposure for at least one day either at home or in their workplace were classified as exposed to secondhand smoke.

Weight, height and blood pressure were measured at the household by a trained professional through standard methods. We used measured weight and height to calculate body mass index (BMI) as kg/m^2^. Participants were classified into four groups: underweight (BMI < 18.5), normal weight (18.5 ≤ BMI < 25), overweight (25 ≤ BMI < 30), and obese (BMI ≥ 30).

Respondents were also asked, “Does your health now limit you in doing vigorous activities, such as running, lifting heavy objects, or participating in strenuous sports?”

To assess clinician-diagnosed asthma, respondents were asked, “Has a doctor or other health professional ever told you that you had asthma, otherwise known as reactive airway disease?” Similar questions were asked for chronic obstructive pulmonary disease (COPD), congestive heart failure (CHF) and type 1 diabetes mellitus.

Participants were asked, “During the past 30 days, have you had wheezing or whistling in your chest?” and “Did you cough on most days for 3 consecutive months or more during the past year?” as important symptoms of respiratory diseases; we did not use these questions as alternative for diagnosis of respiratory diseases.

Those who reported a diagnosis of asthma were asked about treatment (“During the past 30 days, or since your diagnosis, have you ever taken medication for this condition?”), asthma attack (“During the last 12 months did you have an attack of asthma?”), worsening of symptoms (“During the past 12 months, has your asthma gotten worse?”), and visiting a hospital, or emergency room because of worsening asthma symptoms (“During the past 12 months, how many times have you visited the hospital or emergency room because of worsening asthma symptoms?”).

In all analyses, 95 % was used as the level of significance. We used a backward elimination multivariate logistic regression model to measure association between the outcome variables of asthma and different factors, including sex, age, education, smoking, secondhand smoke exposure, BMI, type 1 diabetes and hypertension. All independent variables were first included in the model. Then variables were removed based on a Wald chi-square test for analysis of effect; variables were removed one by one based on the significance level of their effect on the model, starting with the variable with the highest *p* > 0.5, until all variables kept had a *p* ≤ 0.5 for at least one of their subcategories in the final model.

Of the 10,735 completed interviews, there were 74 missing values for asthma, 310 missing values for limited vigorous activity, 115 missing values for diagnosed diabetes status, 29 missing values for smoking status, 1572 missing values for secondhand smoking exposure, 398 missing BMI values, 33 missing marital status values, and 20 missing educational level values. We excluded missing variables from our analyses.

We used Stata 13.1 for Windows (StataCorp LP, TX, USA) for the analyses and to account for the complex sampling design.

## Results

**Fig. 1:**
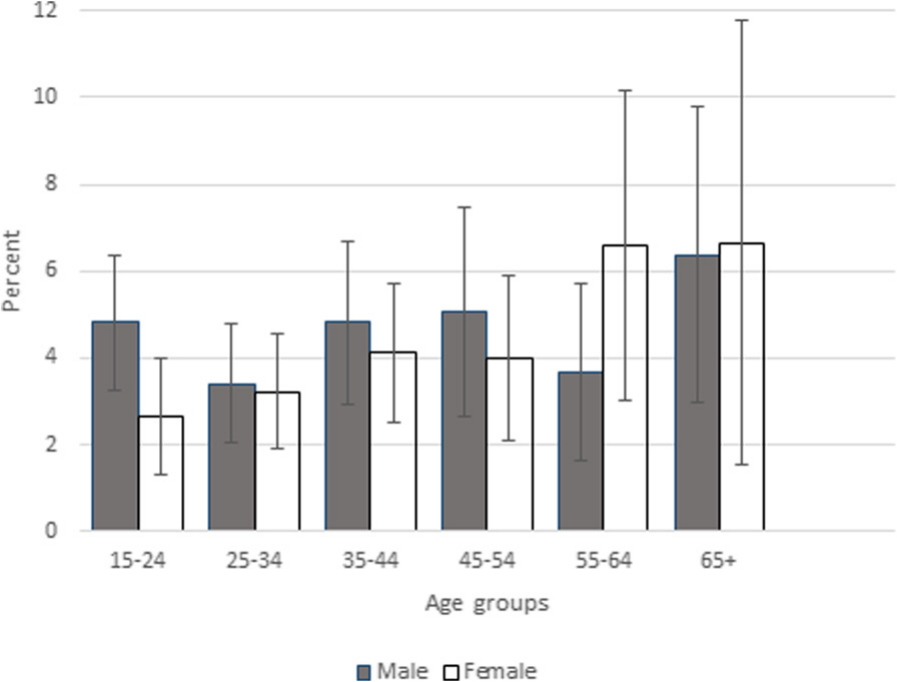
Prevalence (and 95 % confidence intervals) of asthma by sex and age group, Saudi Arabia, 2013

**Fig. 2:**
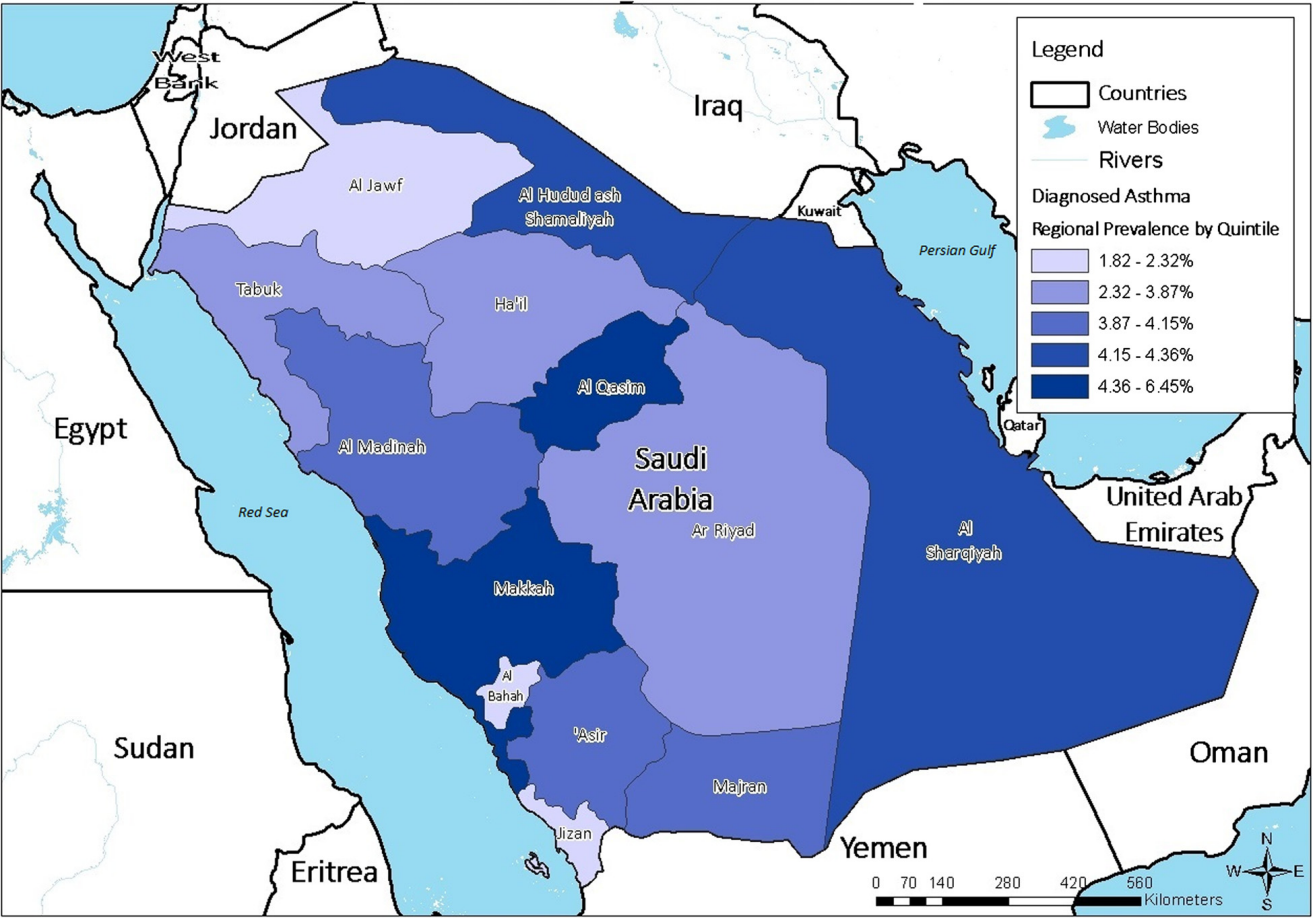
Prevalence range of asthma in different regions of Saudi Arabia, 2013

A total of 12,000 households were originally contacted, and 10,735 participants completed the Saudi Health Interview Survey between April and June 2013 (response rate: 89.4 %). Prevalence of asthma was 4.05 % (95 % CI: 3.54–4.62 %); we estimated there are 525,794 Saudis aged 15 years or older with asthma in KSA. Figure 1 shows the prevalence of asthma by sex and age groups. There was a slightly increasing trend from the third decade of life in both sexes. However, the prevalence was not statistically significant between age-sex subgroups. Prevalence of asthma varied slightly by KSA regions (Fig. 2).

A logistic regression model was used to assess association of different socio-demographic and health factors with asthma (*p* < 0.001). Individuals with college or higher education were less likely to have asthma than individuals with primary education or less. Asthma was associated with being a former smoker (Table 1).

**Table 1:** The prevalence of asthma in relation to socio-demographic and health characteristics in Saudi adults, 2013

Factor	Categories	Total number (Weighted %)	Prevalence		p^a^	AOR	95 % CI
			Cases (Percent)	SE			
Sex	Male	5224 (50.71)	210 (4.53)	0.42	0.08	Ref	-
	Female	5437 (49.29)	180 (3.56)	0.38		0.79	0.57–1.08
Education	Primary or less	3251 (26.27)	150 (5.23)	0.61	0.03	Ref	-
	Elementary/high	4755 (51.92)	149 (3.76)	0.40		0.72	0.52–1.01
	College or higher	2637 (21.80)	91 (3.40)	0.47		0.59	0.39–0.87
Smoking status	Never smoker	8837 (84.06)	315 (3.90)	0.30	0.04	Ref	-
	Former smoker	453 (3.733)	29 (7.47)	1.75		2.01	1.14–3.55
	Current smoker	1347 (12.21)	44 (4.13)	0.80		1.04	0.65–1.66
Secondhand smoking	Unexposed	7048 (76.75)	260 (4.16)	0.36	0.84	Ref	-
	Exposed	2122 (23.25)	93 (4.61)	0.65		NA	-
Body mass index (kg/m^2^)	18.5–24.9 (Normal)	2973 (32.72)	77 (2.81)	0.41	<0.001	Ref	-
	<18.5 (Underweight)	447 (6.505)	18 (5.88)	1.59		2.00	1.03–3.88
	25–29.9 (Overweight)	3431 (29.72)	114 (3.11)	0.41		1.06	0.69–1.63
	30 or more (Obese)	3810 (31.05)	179 (5.90)	0.60		1.98	1.33–2.95
Type 1 diabetes	No	10,422 (98.87)	62 (6.66)	1.09	0.002	Ref	-
	Yes	150 (1.129)	328 (3.85)	0.29		0.35	0.08–1.49
Blood pressure	Normal	4767 (49.58)	161 (3.79)	0.40	0.04	Ref	-
	Pre-hypertension	3663 (31.54)	126 (3.66)	0.45		0.87	0.61–1.24
	Stage I hypertension	1196 (8.933)	65 (6.52)	1.18		1.37	0.85–2.23
	Stage II hypertension	1035 (9.951)	38 (4.43)	0.94		1.23	0.72–2.09

Age was removed during the backward elimination (*p* > 0.5)

*SE* standard error, *AOR* adjusted odds ratio, *NA* not applicable because the subgroup was removed from the model during the backward elimination, *Ref* reference group

^a^p for chi^2^ unadjusted tests

About 15.5 % (95 % confidence interval [CI]: 10.7–21.6) of individuals with asthma reported that they had not taken any medication for asthma at all; 49.4 % (95 % CI: 42.4–56.4 %) were under treatment at the time of survey, and 35.3 % (95 % CI: 28.9–42.1 %) reported taking medication previously, but not at the time of the survey.

Around 76.7 % of individuals with asthma (95 % CI: 70.6–82.0 %) had experienced an asthma attack during the last 12 months, and 31.2 % (95 % CI: 27.4–38.4 %) believed that their asthma had gotten worse in the past 12 months. Logistic regression models were used to find associated factors with asthma attacks and emergency room/hospital visits. Both models were statistically significant (*p* < 0.001). Asthma attack was less frequent in older patients. Current use of medication for asthma was highly associated with asthma attacks (odds ratio [OR] = 9.14, 95 % CI: 3.29–25.38). Asthma attack was also more frequent in individuals who were exposed to secondhand smoke (Table 2).

**Table 2:** Factors associated with asthma attack and visiting an emergency room/hospital because of asthma during the past 12 months in individuals with asthma, Saudi Arabia, 2013

Factor	Categories	Asthma attack		Visit to ER/hospital	
		AOR	95 % CI	AOR	95 % CI
Age (decade)		0.76	0.59–0.96	NA	-
Sex	Male	Ref	-	Ref	-
	Female	0.77	0.36–1.63	NA	-
Education	Primary or less	Ref	-	Ref	-
	Elementary/high	0.66	0.25–1.75	0.45	0.22–0.96
	College or higher	0.37	0.14–1.02	0.26	0.11–0.62
Medication for asthma	Never used	Ref	-	Ref	-
	Current use	9.14	3.29–25.38	2.53	1.01–6.34
	Previous use	1.74	0.71–4.25	0.64	0.24–1.70
Smoking status	Never smoker	Ref	-	Ref	-
	Former smoker	0.52	0.17–1.61	0.39	0.15–0.97
	Current smoker	NA	-	NA	-
Secondhand smoke exposure	Unexposed	Ref	-	Ref	-
	Exposed	2.17	1.05–4.45	1.37	0.62–2.99
Body mass index (kg/m^2^)	18.5–24.9, Normal	Ref	-	Ref	-
	<18.5, Underweight	NA	-	3.95	0.83–18.92
	25–29.9, Overweight	2.28	0.84–6.18	NA	-
	30 or more, Obese	3.01	1.34–6.78	NA	-

*AOR* adjusted odds ratio, *NA* dropped from the model during the backward elimination, *Ref* reference group

During the past 12 months, 61.6 % (95 % CI: 54.4–68.4 %) had visited a hospital or emergency room because of worsening asthma symptoms. That was less likely for individuals with higher education and former smokers (Table 2). About 72 % (95 % CI: 63.5–78.9 %) who had an asthma attack were referred to a hospital or emergency room. Those with asthma were more likely to miss days of work compared to those without (3.8 vs. 2.6 days in the past year, *P* = 0.024).

Those with asthma were more likely to report wheezing or whistling in the chest during the last 30 days compared to others, 58.5 % (95 % CI: 51.4–62.3 %) and 3.7 % (95 % CI: 3.2–4.2 %), respectively. Furthermore, they were more likely to report cough on most days for three consecutive months or during the past year (9.8 % vs. 1.9 %), limitations for vigorous physical activity (55.3 % vs. 34.1 %), COPD (1.7 % vs. 0.13 %), and congestive heart failure (1.8 % vs 0.25 %). The associations between asthma and vigorous physical activity, COPD and congestive heart failure remained significant after adjustment of socio-demographic factors, BMI and smoking in statistically significant logistic regression models (Table 3).

**Table 3:** Association of limited vigorous activity, chronic obstructive pulmonary disease and congestive heart failure with asthma, KSA, 2013

Factors	Categories	Model 1		Model 2		Model 3		Model 4	
		AOR	95 % CI	AOR	95 % CI	AOR	95 % CI	AOR	95 % CI
Age (decades)		0.90	0.79–1.03	NA	-	NA	-	0.88	0.77–1.01
Sex	Male	Ref	-	Ref	-	Ref	-		-
	Female	0.68	0.49–0.95	0.78	0.56–1.08	0.78	0.56–1.08	0.68	0.48–0.95
Education	Primary or less	Ref	-	Ref	-	Ref	-		-
	Elementary/high	0.77	0.52–1.12	0.72	0.51–1.01	0.7	0.5–0.98	0.74	0.50–1.09
	College or higher	0.65	0.42–1.01	0.6	0.4–0.91	0.6	0.4–0.9	0.66	0.42–1.02
Limited vigorous activity	No	Ref	-	NI	-	NI	-		-
	Yes	2.60	1.78–3.79	NI	-	NI	-	2.48	1.69–3.62
Chronic obstructive pulmonary disease	No	NI	-	Ref	-	NI	-		-
	Yes	NI	-	11.38	4.33–29.93	NI	-	9.53	2.90–31.35
Congestive heart failure	No	NI	-	NI	-	Ref	-		-
	Yes	NI	-	NI	-	5.07	1.52–16.86	2.70	0.63–11.55
Smoking status	Never smoker	Ref	-	Ref	-	Ref	-		-
	Former smoker	1.94	1.09–3.43	2.01	1.14–3.54	2.06	1.17–3.61	1.97	1.10–3.53
	Current smoker	1.05	0.66–1.69	1.05	0.65–1.69	1.05	0.65–1.69	1.05	0.64–1.7
Body mass index (kg/m2)	18.5–24.9, Normal	Ref	-		-		-		-
	<18.5, Underweight	1.98	1–3.92	2.02	1.02–3.99	2.12	1.1–4.11	2.07	1.04–4.1
	25–29.9, Overweight	1.09	0.70–1.70	1.07	0.69–1.66	1.12	0.73–1.73	1.16	0.74–1.81
	30 or more, Obese	2.01	1.34–3.03	2.09	1.42–3.08	2.14	1.45–3.15	2.13	1.41–3.22

*AOR* adjusted odds ratio, *NA* dropped from the model during the backward elimination, *NI* not included in the model, *Ref* reference group

## Discussion

Our study revealed relatively low prevalence of asthma in the Kingdom of Saudi Arabia. Our findings showed that those diagnosed with asthma in KSA do not have good control over their condition; incidence of asthma attacks and referral to hospitals for asthma management were considerably high. Moreover, our data showed that many of these individuals do not take appropriate medical care in order to control their asthma. This finding is of great importance as medical care is free in KSA.

The KSA Ministry of Health launched several programs to improve asthma awareness. The Asthma Insights and Reality in the Kingdom of Saudi Arabia (AIRKSA) was launched in 2008 to assess the level of asthma control among asthmatics [10]. Another asthma initiative was launched in 2009 to promote best practices in asthma management [4]. Although it is too early to tell the impact of these programs, clearly there is a need for a comprehensive program for early diagnosis and appropriate management of asthma for children and adults. Indeed, good management of asthma has a positive impact on quality of life of patients [11].

Our study showed that 62 % of asthma patients reported an emergency room visit, which was very close to the 65 % reported by AIRKSA. The numbers are not directly comparable, but the fact that they are close, and very high, calls for immediate attention to asthma in KSA. The annual rate of emergency room visits by people with asthma in European countries is around 11 % [10]. This points to a lack of control of asthma in KSA, which creates a demand for emergency care. This is not sustainable, and programs to reverse this trend are urgently needed.

There are very few studies on the prevalence of asthma in adults in the countries of the region. Estimates are available for physician-diagnosed asthma in Turkey, from 5.1 to 6.2 % in urban areas and from 3.5 to 10.8 % in rural areas [12, 13]. Mohammad reported asthma in 13.1 % of individuals visiting primary health care facilities, outpatient facilities, or emergency departments in Syria [14]. Mahboub reported an asthma prevalence of 12.1 % (95 % CI: 10.4–14.1 %) for all ages in the United Arab Emirates based on European Community Respiratory Health Survey screening criteria [15].

Our study has some limitations. First, our data are cross-sectional and hence we cannot assess causality. Second, many of our behavioral data, such as those related to smoking, are self-reported and subject to recall and social desirability biases. Third, in this study, we report cases of self-reported diagnosed asthma. Milder cases are more likely to be undiagnosed or not reported by the participants. This might be related to high frequency of asthmatic attacks and referral to hospitals in our cases. Fourth, our survey did not collect information on family history of asthma or personal history of allergic rhinitis or hay fever. However, our study is based on a large national sample with rigorous data-collection methods and near-real-time data quality monitoring through the whole survey period.

It is not clear why many patients in Saudi Arabia do not receive proper treatment when health care and medications are free for Saudi nationals. There is an urgency to reduce the financial and human burden of preventable and controllable diseases and risk factors in KSA. Patient education is needed to reduce the burden of asthma in KSA. The primary focus should be on individuals with asthma (and their families) for improving the quality of disease management [16]. Moreover, a national plan to inform the general public about the importance of early diagnosis and management of asthma is needed. We have previously shown that Saudis do not seek preventive care; [17] therefore these programs should be promoted as generally increasing Saudis’ health, not as specific preventive activities. Also, there are specific instructions to reduce allergic asthma and occupational asthma through avoiding or reducing exposure to allergens [16, 18–20]. Finally, programs to educate physicians and medical assistants about appropriate ways to message the importance of preventive care and adequate follow-up for asthma are needed. Since different studies have emphasized low adherence by health care providers to clinical guidelines for asthma in KSA, new programs should include methods for improving this adherence [4, 21, 22].

Our finding of high rates of asthma among former smokers deserves further attention. Indeed, in a cross-sectional study, we cannot detect temporality of getting asthma and quitting smoking, but the fact that the relationship of asthma with former smoking is stronger than with current smoking has been shown in other studies as well. Some individuals stop smoking because of the onset of respiratory symptoms and are later diagnosed with asthma, and some individuals quit smoking because of diagnosed asthma [23–25]. The association of smoking and asthma is strong and well documented [11, 25]. On the other hand, we pooled all current smokers without considering their history of smoking over time, which might influence the analysis results. Other findings of our survey revealed an increasing trend in cigarette smoking among men and shisha smoking among men and women in KSA [26]. Therefore, smoking prevention programs should be strengthened in KSA in order to reduce the burden of all diseases caused or aggravated by tobacco products (including asthma). Based on our data, exposure to secondhand smoking was associated with asthma attacks, which underscores the importance of tobacco-free settings.

There is no consensus on the association of asthma with a desert environment. Some studies did not find a relationship between sand storms and asthma prevalence or aggravation, while others consider these storms important risk factors for chronic respiratory disease and severity of asthma [27, 28]. Based on the report of the Global Initiative for Asthma (GINA) Program, Tunisia, Ethiopia and Algeria, from a relatively close geographical region to KSA, are among the countries with the lowest prevalence of current symptoms of asthma in adults (all less than 5 %), while some of the high-income Commonwealth countries, such as Wales, Australia, Scotland, Republic of Ireland and Canada, have the highest prevalence estimates (all more than 25 %) [29]. In this study, we did not observe noticeable differences in the prevalence of asthma between different official governmental regions of KSA.

## Conclusion

Saudi Arabia has a relatively low prevalence of diagnosed asthma; however, many of the patients with known asthma are not under good control. Our study calls for programs to inform patients about the importance and proper means to control their condition. Moreover, our study calls for programs to educate the Saudi public about the importance of preventive strategies, early diagnosis and proper management of asthma.
